# Evaluation of accuracy, filter performance, and durability among capnography sampling lines: a bench study

**DOI:** 10.1007/s10877-025-01346-3

**Published:** 2025-08-28

**Authors:** Terry L. Jones, Denis Glozman, Harold Julius Augustus Oglesby, Jan Paul J. Mulier

**Affiliations:** 1 School of Nursing, Virginia Commonwealth University, Richmond, VA USA; 2 Research and Development, Acute Care and Monitoring, Medtronic, Jerusalem, Israel; 3 Pulmonary Medicine, St. Joseph’s/Candler Health, Savannah, GA USA; 4 Department of Anesthesiology, Faculty of Medicine and Health Sciences, Ghent University, Ghent, Belgium

**Keywords:** Respiratory compromise, Respiratory depression, Capnograph, End-tidal carbon dioxide, Monitoring

## Abstract

The aim of this bench study was to assess performance between currently available sidestream capnography sampling lines claiming compatibility with a Microstream™ enabled monitor system. A series of bench tests were performed on a selection of commercially available sampling lines (oral-nasal cannulas, PetCO_2_ masks, and procedural bite blocks) to assess PetCO_2_ accuracy, filter performance and mechanical durability. For each testing procedure, three separate sampling lines were assessed for five repetitions (n = 15 for each product). A 3D printed oral-nasal model connected to a lung simulator was set to two different breathing modes to simulate “normal” and “shallow” breathing. PetCO_2_ accuracy measurements were completed at both breathing modes to simulate a variety of clinical scenarios, including oxygen delivery. Filter performance and mechanical durability were assessed for cannula sampling lines. When evaluating PetCO_2_ accuracy across all sampling line types and breathing conditions, error estimates were lowest for matched-paired sampling lines, compared to cross-paired sampling lines. For oral-nasal cannulas, the matched-paired and three cross-paired performed within the system specification for “normal” breathing conditions. Split design oral-nasal cannulas consistently fell outside specifications under conditions of nasal obstruction. Five cross-paired filters clogged prior to expected threshold, with four demonstrating a clog capacity of less than half the expected value. Almost all sampling lines passed mechanical durability testing. Across all testing conditions, cross-paired sampling lines were observed to fall outside system specifications, suggesting that using sampling lines outside the Microstream™ family may disrupt accuracy of readings when using a Microstream™ enabled capnography monitor.

**Supplementary Information:**

The online version contains supplementary material available at 10.1007/s10877-025-01346-3.

## Introduction

A substantial body of evidence supports the efficacy of capnography monitoring in detecting respiratory compromise and averting associated adverse events [1–4]. Intraoperative capnography monitoring has long been the standard of care for the early detection of respiratory compromise associated with anesthesia [5, 6]. Traditional capnography monitoring technology uses non-dispersive black body infrared radiation (IR) techniques for analysis of exhaled gases and are categorized as either mainstream (non-diverting), with measurements taken directly in-line of air flow or sidestream (diverting), with measurements taken from breath samples that have been routed through a sampling line to be analyzed in monitor [7, 8]. Though mainstream monitoring provides reliable, high-fidelity measurements, its use outside the operating room and intensive care unit has been limited in practice [7, 9]. As sidestream monitoring is perceived to be more manageable, notably in non-intubated patients, its use throughout the rest of the hospital and other care settings is more pervasive [3, 10–12].

Noted limitations of traditional sidestream monitoring systems include delayed response times (up to several seconds) [13], underestimation of end-tidal carbon dioxide (PetCO_2_)PetCO_2_ [14, 15], sensitivity to volume and flow rate making it less accurate in certain patient populations, and condensation in the system that can lead to clogging [16]. In contrast to the IR analysis techniques used by traditional sidestream monitors, Microstream™ technology uses molecular correlation spectroscopy to evaluate PetCO_2_ and solves for some of the aforementioned limitations [9, 17]. The molecular correlation spectroscopy technique emits only CO_2_ specific radiation to permit a lower pathlength that allows for smaller breath samples and lower flow rates [18]. Further, this technology eliminates the need for filtering of breath samples, effectively shortening the delay time, and an integrated water filter eliminates the need for a separate water trap [17]. Even so, the sampling line has the potential to impact measurement accuracy if the tubing interface taints the sample in any way while in transit to the monitor [8, 12, 19, 20]. Air trapping and rebreathing may occur in the sampling line, especially in cases of higher breathing rates, and when turbulent flow is present within the sampling line, due to length, diameter, condensation, or a combination, which may also impact measurements and distort capnography readings. Further, filter blockage and mechanical degradation, such as cracked or loose connections resulting in system leaks, can impede accurate measurement [16, 21]. Relatedly, the fidelity of the breath sample is impacted by the individual sampling line design, dependent on where and how the breath sample is collected in respect to the patient and oxygen delivery system, if applicable [8]. As there are several components that make up a sidesteam monitoring system, it is vital that all components work individually and together as intended to ensure accurate and responsive measurements.

Though capnography monitoring enjoys broad endorsement as the standard of care for surveilling patients at risk for respiratory compromise [22–27], the adoption of capnography monitoring comes with associated costs. Equipment expenses contribute to hospital supply budgets, which rank second only to labor expenses. Supply costs typically account for 15–40% of operating budgets and have shown more significant increases than labor expenses over the past decade [28]. Consequently, supply chain managers seek opportunities to reduce these costs, often through a product evaluation process that involves a comparative analysis of cost and value. Sidestream capnography supplies include the monitoring unit housing the sensor and the sampling line responsible for extracting and transporting the gas sample from exhaled breath. Several factors, including oxygen dilution, filter quality, and mechanical stability of the sampling line can influence the accuracy of capnography readings, durability of the lines, and cost. Therefore, when assessing the value of capnography, careful consideration of the performance of the sampling line is essential. The aim of this bench study was to evaluate performance of currently available sidestream capnography sampling lines claiming compatibility with a Microstream™ enabled monitoring system by assessing accuracy, filter performance, and mechanical durability based on system specifications.

## Methods

A series of bench tests compared the performance of sidestream sampling lines (Table 1) commonly used with spontaneously breathing patients under various breathing conditions. Three categories of sampling line products were evaluated to reflect the application of capnography monitoring: oral-nasal cannulas, PetCO_2_ masks, and procedural bite blocks. All tested sampling lines feature an oxygen delivery option, enabling assessment of error associated with oxygen dilution.

**Table 1:** Sampling lines tested

Sampling Line Manufacturer	Type	Product description	Reference number	Rise time	EtCO_2_ accuracy	Filter performance	Pull test	Leak test
Microstream™ Advance Filter Line Medtronic	Oral Nasal Cannula	Adult Oral-Nasal CO_2_ sampling line; O_2_ delivery up to 5 lpm; CO_2_ line & O_2_ line 2 m Microstream Advance	MVAO	X	X	X	X	X
Salter Labs	Oral Nasal Cannula	Adult O_2_/ETCO_2_ Filtered Oral/Nasal Divided Cannula. Oxygen delivery up to 4 lpm	4MSF1-7-6	X	X	X	X	X
Comfort Soft-Plus® Westmed	Oral Nasal Cannula	CO_2_/O_2_ Oral/Nasal Cannula, Adult Oral/Nasal Cannula, 7’ CO_2_/CO_2_ with reflective Luer and Comfort Soft Plus Tubing	0917	X	X	X	X	X
NomoLine-O LH Masimo	Oral Nasal Cannula	NomoLine-O LH Adult Nasal/Oral CO_2_ Cannula with O_2_ up to 5 lpm	4459	X	X	X	X	X
PRO-Breathe™ PROACT	Oral Nasal Cannula	Adult with O_2_ delivery up to 5 lpm	PBMS04561	X	X	X	X	X
VentFLO™ SunMed®	Oral Nasal Cannula	Adult Nasal ETCO_2_/O_2_ Nasal/Oral Cannula + Scoop	5707F-SE	X	X	X	X	X
Curaplex^1^	Oral Nasal Cannula	Adult Nasal ETCO_2_/O_2_ Nasal/Oral Cannula + Scoop	301-5707F-SE	X	X	X	X	X
MicroFilter MedLine	Oral Nasal Cannula	Microfilter Oral/Nasal Adult CO_2_ Cannula with O_2_delivery up to 5 lpm	HCS4607MO	X	X	X	X	X
Dual Cannula Flexicare	Oral Nasal Cannula	Dual Adult Oral Nasal Cannula 7’ O_2_ delivery/CO_2_ Monitoring, Filter, “Microstream Type” Luer with O_2_ delivery up to 6 lpm	032-10-180U	X	X	X	X	X
DualGuard™ Flexicare	Procedural Bite Block	Adult Endoscopy Mouthpiece 14ft O_2_ delivery and CO_2_ monitoring	032-10-870U	–	X	–	–	–
Microstream™ Advance Medtronic	Procedural Bite Block	Adult/Intermediate CO_2_ Oral/Nasal sampling and bite block set with O_2_ tubing. O_2_ delivery up to 10 lpm	012530	–	X	–	–	–
CapnoVue® Airlife	EtCO_2_ Mask	Adult mask with oral scope access and thread grip. 7’ tubing. No more than 4 lpm O_2_ delivery rate	4737TG-7-0	–	X	–	–	–
Oxy2Pro™ with Microstream™ connector Southmedic	EtCO_2_ Mask	Adult mask with oral scope access. oxygen delivery from 5–15 lpm	PRM-2110-MDT	–	X	–	–	–
Procedural Oxygen Mask™ (POM) Stryker	EtCO_2_ Mask	Adult oxygen mask with multi-port design to accommodate a wide range of scopes, probes, and tubes	1001-MSA	–	X	–	–	–
PrO2 Mask Curaplex®	EtCO_2_ Mask	Adult endoscopy oxygen mask with dual oral and nasal ports and O_2_ delivery rate of 10–15 lpm	301-PrO2LTEZ-MSA	–	X	–	–	–

X indicates which tests were completed

^1^Curaplex cannula appeared identical to the VentFLO cannula, including name on package, though the reference numbers differed

The Capnostream™ 35 Portable Respiratory Monitor (Medtronic, Minneapolis, MN), a Microstream™ enabled capnography system, was used for data collection. The monitoring technology and accuracy specifications of this monitor are the same, or similar, to other Microstream™ enabled capnography systems so the results observed in this study would be expected to be relevant to these other systems. Although all selected products claim compatibility with Microstream™ enabled capnography systems, they are produced by different manufacturers and not validated by the manufacturer of Microstream™ capnography system. The matched pair condition for oral-nasal cannulas was the Microstream™ Advance Filter Line, for procedural bite blocks was the Microstream™ Advance bite block filter line, and for PetCO_2_ masks was the Oxy2Pro™* with Microstream™ connection. All other pairings were considered crossed-pair conditions.

### Testing procedures

**Fig. 1:**
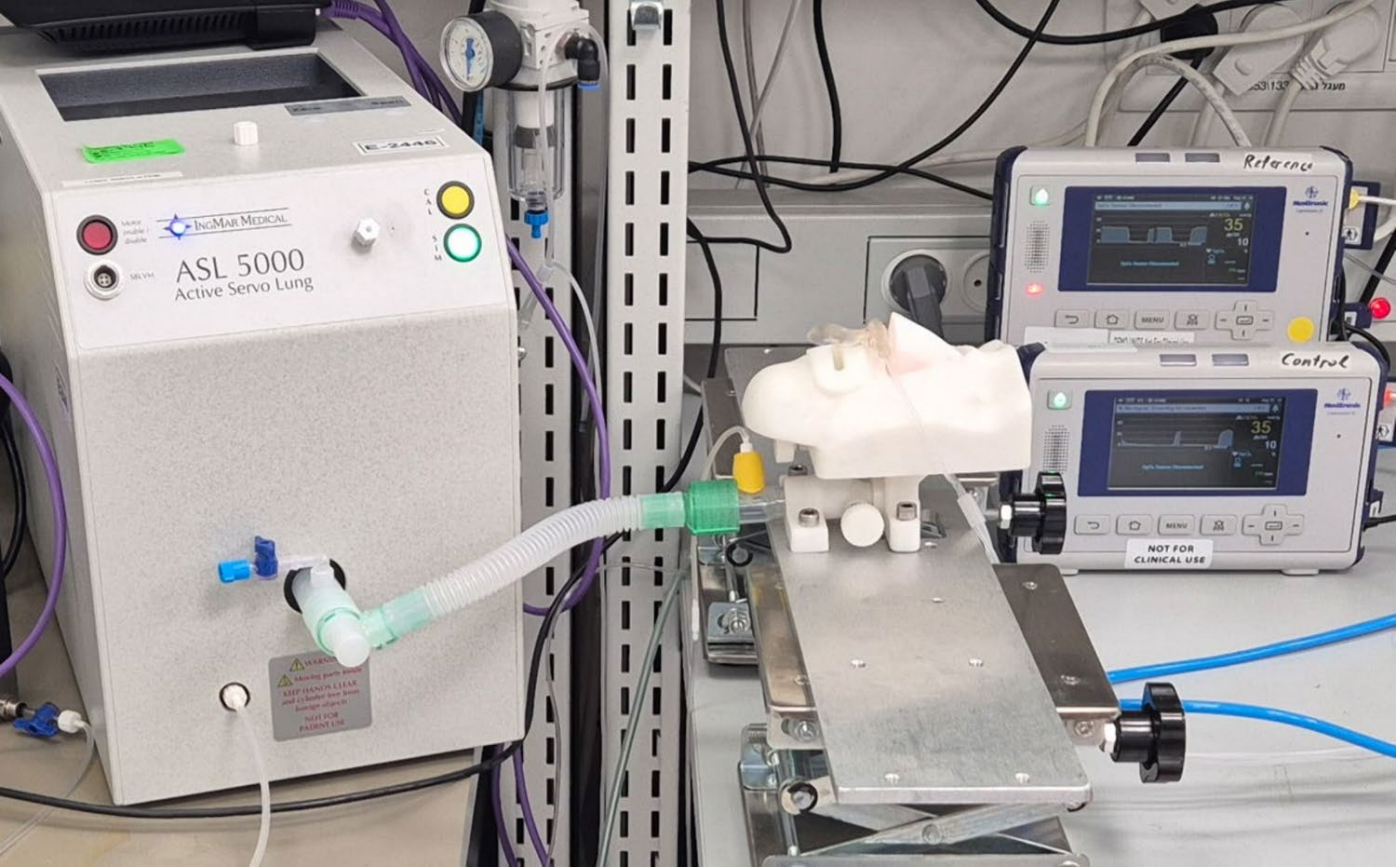
Second testing system set-up, utilized to evaluate PetCO_2_ accuracy during both nasal and oral breathing, with and without oxygen, and under normal and shallow breathing conditions. A lung simulator, connected to a 3D printed oral-nasal cavity, was used to generate natural breathing airflow patterns, regulate reference CO_2_, and administer supplemental oxygen. Sampling lines were attached to the 3D printed oral-nasal cavity and the capnography monitoring system for testing

A series of tests were performed to assess accuracy, filter performance, and mechanical durability following established capnography testing protocols [29]. All sampling lines and PetCO_2_ masks used for this study were designed for adult patients, so adult settings and physiological parameters were utilized. Breathing conditions for testing were simulated using two different mechanical systems. The first system employed was a calibrated solenoid-based breathing simulator capable of delivering controlled carbon dioxide (CO_2_) concentrations across a full range of breathing rates with a square wave pattern, allowing for the assessment of PetCO_2_ accuracy under extreme conditions, thereby testing the equipment’s limits. In this study, the simulator delivered inspired air containing 5% CO_2_ without supplemental oxygen, resulting in a predicted PetCO_2_ value of 36 mmHg. The simulator was set to deliver respiratory rates ranging from 10 breaths per minute (bpm) (normal rate) to 80 bpm (extreme rate) in 10 bpm step increments. This system facilitated accuracy assessment based on rise time and PetCO_2_, as described below. The second system involved a lung simulator capable of generating airflow patterns resembling natural breathing through nasal or oral cavity. This simulator aimed to mimic a capnograph pattern expected in spontaneously breathing patients. The oral-nasal cavity was created based on anatomical features as derived from medical textbooks and CT scans [30–32]. Subsequently, the cavity was modeled, 3D printed, and connected to the ASL5000® lung simulator (IngMar Medical, Pittsburgh, PA) to regulate reference CO_2_ and administer supplemental oxygen (Fig. 1). This system was utilized to evaluate PetCO_2_ accuracy during both nasal and oral breathing, with and without oxygen, and under normal and shallow breathing conditions as described below.

### Accuracy reflected by rise time

Accurate PetCO_2_ assessment requires analysis of clearly differentiated breath samples [29]. When exhaled gases are mixed—which could be due to system dead space, high flow rate, high respiratory rates, or a combination—breaths cannot be distinguished, making it impossible to measure the true PetCO_2_ level [8, 19]. Capnography systems differentiate breathing cycles based on patterns of change in CO_2_. A transition from increasing to decreasing CO_2_ values indicates the end of a breathing cycle. Delay in detecting this transition can hamper signal integrity resulting in distorted waveforms and inaccurate readings.

**Fig. 2:**
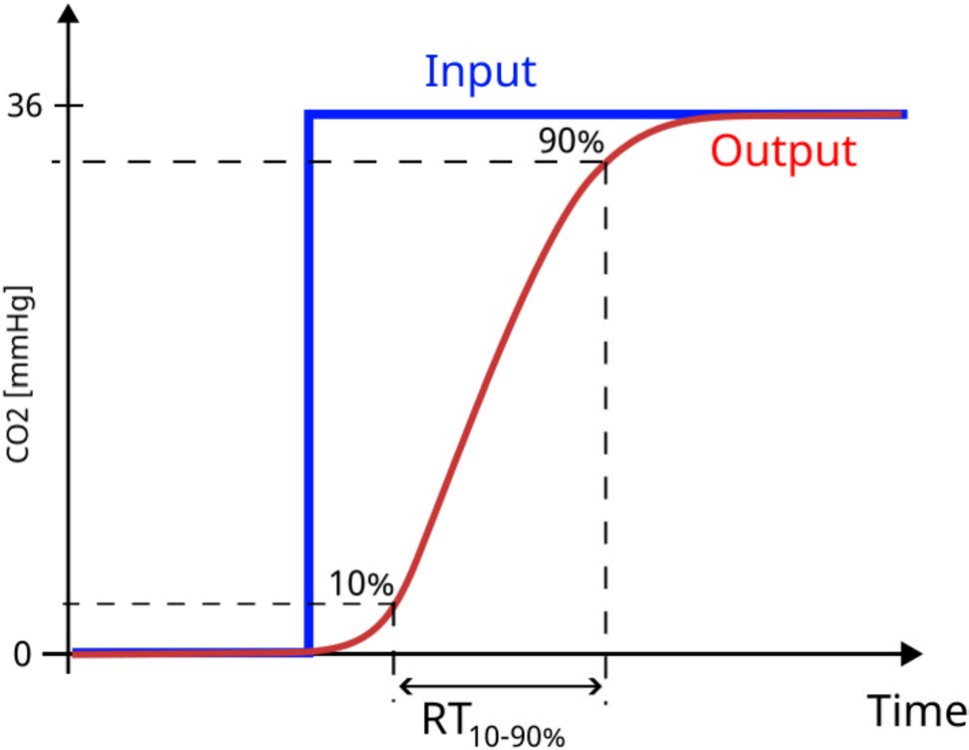
Schematic illustration of rise time

The ability to rapidly detect this transition is quantified by an indicator called rise time. Rise time is defined as the time taken for a measured CO_2_ value to increase from 10 to 90% of the peak PetCO_2_ value (Fig. 2) [33]. Rise time is affected by the pneumatic structure of the whole sampling system such as tubing dead space, airflow, and filter design [29]. Short rise times are optimal as they indicate the capacity to detect the rate of change necessary for breath differentiation. Capnography monitors function best within established rise time parameters. Sampling lines that have rise times outside these parameters are considered incompatible with the system. Rise time was assessed at breathing rates between 10 and 80 bpm, at 10 bpm increments, with nasal only breathing, no nasal obstructed, and no supplemental oxygen. The square wave of CO_2_, was sampled by the capnograph with each oral-nasal cannula sampling line attached. The change in CO_2_ level at 10% and 90% of PetCO_2_ were evaluated, and the time taken between these points was determined. Three separate cannulas from each of the nine product lines were tested five times, for a total of 15 measurements taken for each cannula type. The current specification of rise time for the capnograph system used for testing is up to 190 ms for a 2-m sampling line, according to the Instructions For Use (IFU). Rise times above the system specified criteria were categorized as outside of specifications, (Supplementary Table 1). As the accuracy of the entire system depends on the performance of each component individually and all components together. Therefore, components performing outside the stated system specifications may impact measurement fidelity of the entire system.

### Accuracy as reflected by PetCO_2_

The accuracy of PetCO_2_ was evaluated on all product categories, including oral-nasal cannulas, PetCO_2_ masks, and procedural bite blocks. PetCO_2_ accuracy was defined as the absolute difference between the predicted PetCO_2_ level of 36 mmHg (5% reference CO_2_), and the level recorded on the capnography monitor. Two distinct breathing modes were used during the assessments. The first mode, termed “normal breathing”, simulated a respiratory rate of 10 bpm and a tidal volume of 450 mL. The second mode, termed “shallow breathing”, simulated a respiratory rate of 20 bpm and a tidal volume of 300 mL. For the oral-nasal cannulas, accuracy measurements were completed for the following conditions at both breathing modes: (1) oral breathing only without supplemental oxygen, (2) nasal breathing only with left nostril obstructed and no supplemental oxygen, (3) nasal breathing only with right nostril obstructed and no supplemental oxygen, (4) nasal breathing only with no nasal obstructed with no supplemental oxygen, and then with 5 L per minute (l pm) supplemental oxygen (or at maximal flow specified by the manufacturer).

For the procedural bite blocks, PetCO_2_ accuracy was assessed under both breathing conditions during oral only and nasal only breathing conditions with supplemental oxygen levels of 0, 5, and 10 lpm. For the PetCO_2_ masks, accuracy measures were assessed for both breathing conditions during oral only and nasal only breathing conditions with supplemental oxygen levels of 0, 4, and 15 lpm. Assessment of accuracy during nasal obstruction was not relevant for procedural bite blocks or PetCO_2_ masks. According to the IFU of the Microstream™ system used for testing, the range of acceptable error up to 80 bpm is ± 2 mmHg [34]. The International Organization for Standardization (ISO) standard for capnography monitoring (ISO 80601–2-55) allows for error up to ± 6 mmHg in PetCO_2_ [35].

### Filter performance

Filter performance for the oral-nasal cannulas was assessed as clog capacity. The Microstream™ system with a sampling line has a pressure threshold of 100 mBar above which the system is no longer able to pump and is considered clogged. Clog capacity is defined as the amount of liquid added to the sampling line before this threshold is met and the filter clogs. Clog capacity was tested up to the 160 μl (up to 8 h). Water drops were introduced into the sampling line at a rate of 1 mL per minute while the pumping of the air sampling was set to a standard rate of 50 ml/min, temperature set to 25 °C, until the pressure in the system rose above 100mBar. Products with a clog capacity less than 160 μl were considered to fail the clog test and have poor filter capacity, limiting their expected lifetime performance.

### Mechanical durability

A pull test, also known as a tensile strength test, was undertaken to evaluate structural integrity and mechanical durability of the sampling line in response to external mechanical forces. Commercial sampling line systems have multiple connections between the cannula, humidifier, oxygen delivery tubes, and the monitor. The number and type of connections vary by product. Loose or weak connections, or complete disconnections at any point, introduce error due to the leakage and dilution of the gas sample before reaching the sensor. The tensile strength of connection points is used to indicate the durability of the sampling line under the dynamic conditions of clinical care which may result in cannulas being pulled and stretched by mobile patients or by providers during transport. Each of the oral-nasal cannula sampling lines was gradually stretched with the max speed of 300 mm/min and a maximum force of 20N (2 kg) while being observed for loose/weak connections or complete disconnections. Two types of leak tests were performed on the oral-nasal cannulas. The first type of leak test was performed to assess for leaks related to the breath sample for PetCO_2_ analysis. This was done to detect any dilution of the sample by the air from outside due to unproper sealing. The second type of leak test was related to the oxygen delivery arm of the cannulas. The O_2_ lines were tested to assess whether all oxygen was delivered through the line without leaks in the tubing or connectors. All measurements were completed using a calibrated manometer in a range of up to 345 mBar (5 psi). The carbon dioxide tube was clamped 5–20 mm from the airway adaptor, system pressure was set to 125 ± 25 mBar. The test valve was closed, and pressure was measured to assess for leaks.

### Statistics

The purpose of this study was to evaluate the accuracy, filter performance, and mechanical durability. Accuracy and filter performance were evaluated based on being within or outside specifications of the Microstream™ capnography system [34]. No direct statistical comparison between products was undertaken. Further, no clinical data was collected, therefore readings were not connected to any clinical endpoints. Descriptive statistics were calculated using Minitab (Minitab, LLC, Lock Haven, PA, USA) and are presented as means and standards deviations.

## Results

### EtCO_2_ accuracy—rise time

**Fig. 3:**
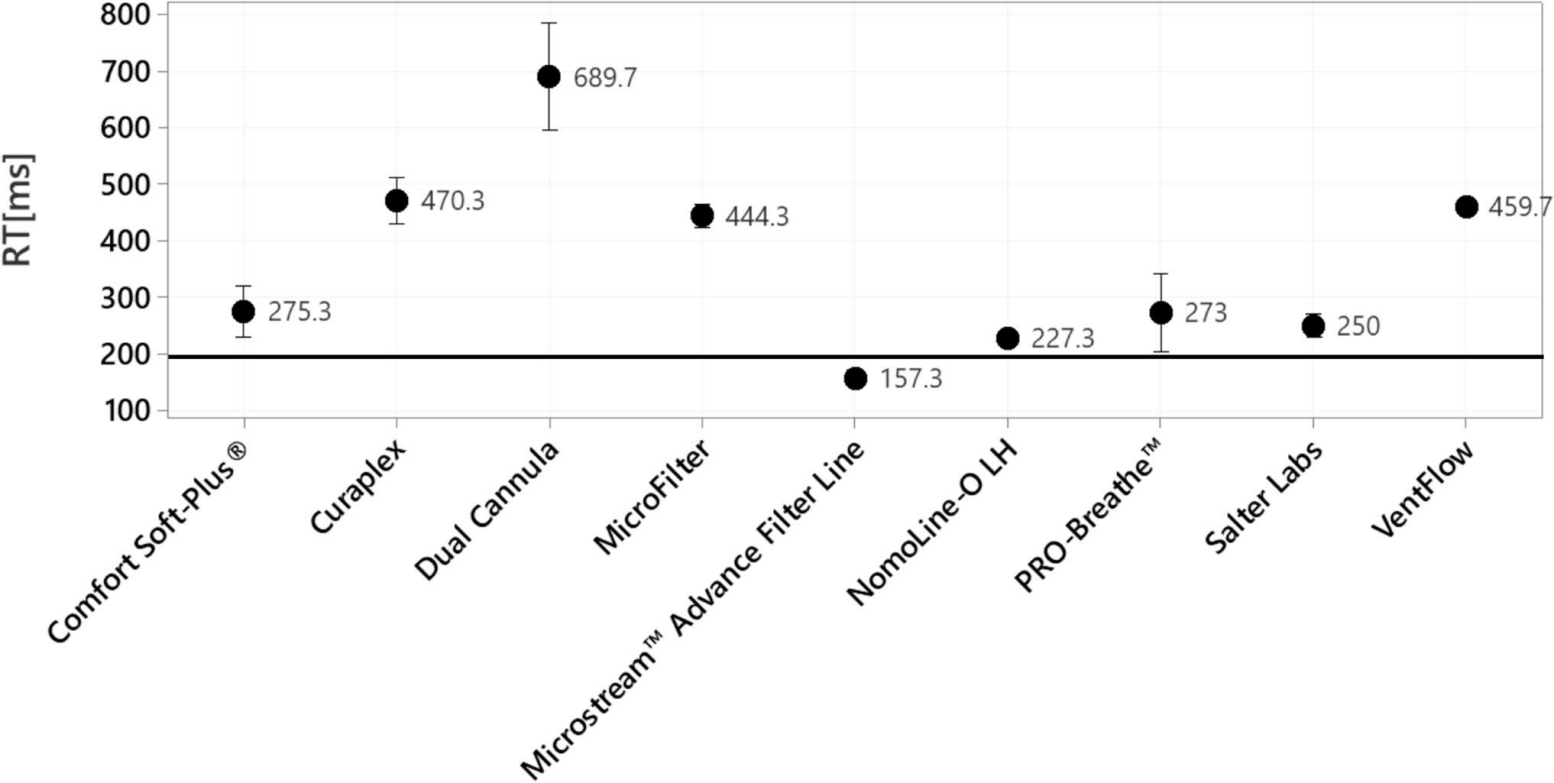
Mean rise time in milliseconds for each cannula. Three separate cannulas were tested five times, for a total of 15 measurements taken for each cannula type. Error bars represent 95% confidence intervals. The black line represents the accepted rise time for the capnograph, according to specifications

The observed average rise time of all oral-nasal cannulas across all breathing rates is presented in Fig. 3. The rise time for the matched pair condition had the lowest rise time and performed within the rise time specification of the capnograph. All cross-paired cannulas performed outside of the rise time specification of the capnograph. Supplementary Fig. 1 illustrates how greater rise time can have a negative impact on the accuracy of the capnography signal.

### CO_2_ accuracy—PetCO_2_

The absolute PetCO_2_ error estimates associated with incremental changes in respiratory rate with square wave breathing pattern are summarized in Table 2. The absolute PetCO_2_ error estimates for all other breathing conditions across sampling lines are summarized in Table 3.

**Table 2:** Accuracy of end-tidal carbon dioxide by sampling line and breathing rates

Cannula type	10 bpm	20 bpm	30 bpm	40 bpm	50 bpm	60 bpm	70 bpm	80 bpm
	Δ EtCO_2_	Δ EtCO_2_	Δ EtCO_2_	Δ EtCO_2_	Δ EtCO_2_	Δ EtCO_2_	Δ EtCO_2_	Δ EtCO_2_
Microstream™ Advance Filter Line	0 ± 0 n = 15, (0, 0)	0 ± 0 n = 15 (0, 0)	0 ± 0 n = 15 (0, 0)	0 ± 0 n = 15 (0, 0)	0 ± 0 n = 15 (0, 0)	0.3 ± 0.6 n = 15 (0, 1)	0.7 ± 0.6 n = 15 (0, 1)	0 ± 0 n = 15 (0, 0)
Salter Labs	0 ± 0 n = 15 (0, 0)	0.7 ± 0.6 n = 15 (0, 1)	0 ± 0 n = 15 (0, 0)	0 ± 0 n = 15 (0, 0)	1.3 ± 0.6 n = 15 (1, 2)	2 ± 0 n = 15 (2, 2)	2 ± 0 n = 15 (2, 2)	1.3 ± 0.6 n = 15 (1, 2)
Comfort Soft-Plus®	0 ± 0 n = 15 (0, 0)	0 ± 0 n = 15 (0, 0)	1.7 ± 0.6 n = 15 (1, 2)	3.3 ± 0.6 n = 15 (3, 4)	5.3 ± 0.6 n = 15 (5, 6)	5 ± 0 n = 15 (5, 5)	5 ± 0 n = 15 (3, 5)	3 ± 0 n = 15 (3, 3)
NomoLine-O LH	0 ± 0 n = 15 (0, 0)	0 ± 0 n = 15 (0, 0)	0 ± 0 n = 15 (0, 0)	1 ± 0 n = 15 (1, 1)	1.7 ± 0.6 n = 15 (1, 2)	1.3 ± 0.6 n = 15 (1, 2)	1 ± 0 n = 15 (1, 1)	0 ± 0 n = 15 (0, 0)
PRO-Breathe™	0 ± 0 n = 15 (0, 0)	0 ± 0 n = 15 (0,0)	0 ± 0 n = 15 (0,0)	1.3 ± 0.6 n = 15 (1, 2)	3 ± 0 n = 15 (3, 3)	2 ± 0 n = 15 (2, 2)	3 ± 0 n = 15 (3, 3)	2.3 ± 0.6 n = 15 (2, 3)
VentFLO™	0 ± 0 n = 15 (0, 0)	0 ± 0 n = 15 (0, 0)	1 ± 0 n = 15 (1, 1)	2 ± 0 n = 15 (2, 2)	3 ± 0 n = 15 (3, 3)	5 ± 0 n = 15 (3, 5)	5.3 ± 0.6 n = 15 (3, 6)	3.3 ± 0.6 n = 15 (3, 4)
Curaplex	0 ± 0 n = 15 (0, 0)	0 ± 0 n = 15 (0, 0)	1 ± 0 n = 15 (1, 1)	2 ± 0 n = 15 (2, 2)	3 ± 0 n = 15 (3, 3)	4 ± 0 n = 15 (4, 4)	5 ± 0 n = 15 (5, 5)	5 ± 1 n = 15 (4, 6)
MicroFilter	0 ± 0 n = 15 (0, 0)	0 ± 0 n = 15 (0, 0)	1 ± 0 n = 15 (1, 1)	2 ± 0 n = 15 (2, 2)	4 ± 0 n = 15 (4, 4)	4.7 ± 0.6 n = 15 (4, 5)	6 ± 0 n = 15 (6, 6)	0 n = 15 (0, 0)
Dual Cannula	1 ± 0 n = 15 (1, 1)	1 ± 0 n = 15 (1, 1)	4.3 ± 0.6 n = 15 (4, 5)	6 ± 0 n = 15 (6, 6)	10 ± 0 n = 15 (10, 10)	9.7 ± 0.6 n = 15 (9, 10)	11.3 ± 0.6 n = 15 (11, 12)	5.6 ± 0.6 n = 15 (5, 6)

Accuracy was defined as difference in measured EtCO_2_ (mmHg) from expected value of 36 mmHg. Data is presented as mean Δ EtCO2 ± standard deviation, n, (min, max). Three separate sampling lines were tested for each product in replicates of five

bpm: breaths per minute, EtCO_2_: end tidal carbon dioxide, mmHg: milimeters of mercury

**Table 3:**
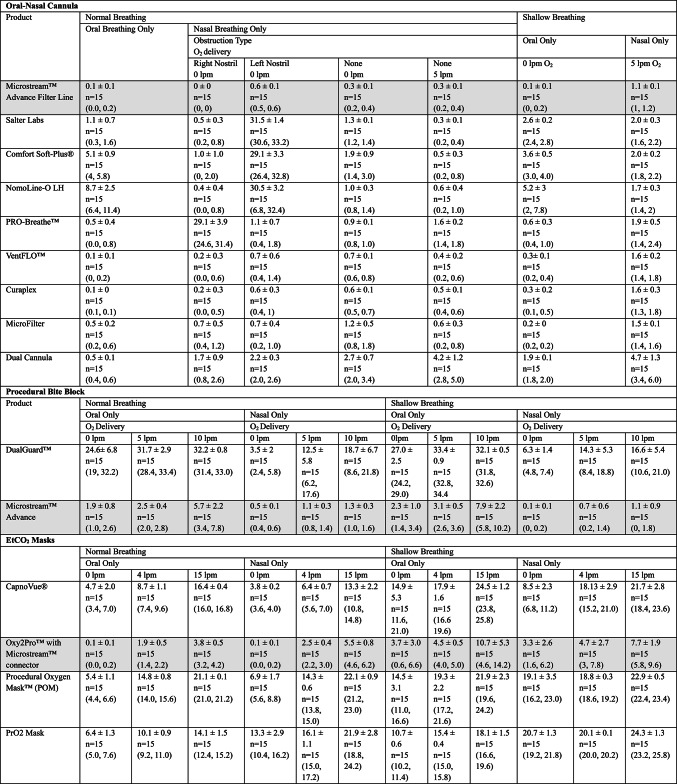
Accuracy of end-tidal carbon dioxide by sampling line and breathing condition

Accuracy was defined as difference in measured EtCO_2_ (mmHg) from expected value of 36 mmHg. Data is presented as mean Δ EtCO2 ± standard deviation, n, (min, max). Three separate sampling lines were tested for each product in replicates of five

bpm: breaths per minute, EtCO_2_: end tidal carbon dioxide, mmHg: milimeters of mecury; l pm: liters per minute

#### Oral-nasal cannulas

Under the square wave breathing pattern without supplemental oxygen, errors in PetCO_2_ readings were generally higher at breathing rates greater than 30 bpm. Only two sampling lines performed within the capnograph’s stated range of acceptability (± 2 mmHg) up to 80 bpm: Microstream™ Advance Filter Line (match paired) and NomoLine-O LH (cross-paired). Among the cross-paired products, four of them (Comfort Soft-Plus®, VentFLO™, MicroFilter, and Flexicare Dual Cannula) reached errors of ≥ 5 mmHg. The Flexicare Dual Cannula exceeded the more liberal ISO error range (± 6 mmHg) at all rates above 30 bpm, reaching error estimates as high as 10 mmHg.

**Fig. 4:**
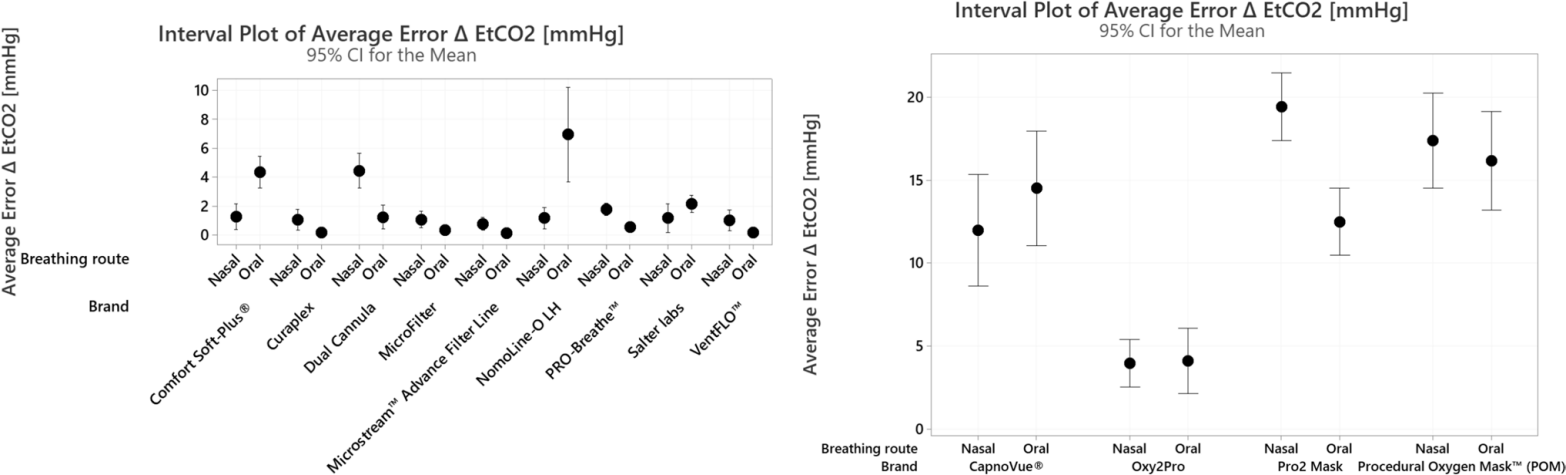
Accuracy of end-tidal carbon dioxide by sampling line and breathing condition. Accuracy was defined as difference in measured PetCO_2_ (mmHg) from expected value of 36 mmHg. Three separate cannulas were tested five times, for a total of 15 measurements taken for each cannula type. Circles represent the mean difference, and the error bars represent 95% confidence intervals

Under conditions simulated by the system with specially designed nasal cavity, three products demonstrated error estimates within the system’s acceptability range across all conditions tested (Fig. 4A). Cannulas with a split design (Salter Labs, Comfort Soft-Plus®, NomoLine-O LH, and PRO-Breathe™) sample from only one nostril demonstrated decreased accuracy under conditions of nasal obstruction. Across all conditions, error estimates were lowest for the matched-pair sampling line.

#### Procedural bite blocks

The matched paired product demonstrated lower error estimates across all conditions compared to the cross paired product (Table 3). The DualGuard™ Flexicare sampling line was associated with an overall absolute error estimate of 29.5 mmHg for “normal” breathing and 30.8 mmHg for “shallow” breathing. In contrast, the Microstream™ Advance sampling line was associated with an overall absolute error estimate of 3.3 mmHg for “normal” breathing and 4.4 mmHg for “shallow” breathing. Greater error was recorded in both product lines during oral only breathing compared to nasal only breathing. Greater error was also recorded in both product lines during “shallow” breathing compared to “normal” breathing. There was no clear effect of supplemental oxygen delivery on error estimates.

#### PetCO_2_ masks

Across all testing conditions, the least amount of error was recorded for the matched-pair product compared to the cross-paired products (Table 3 and Fig. 4B). Reported error during normal breathing conditions with oral only breathing ranged from 2.1 mmHg at 4 lpm (Oxy2Pro) to 21.1 mmHg at 15 lpm (POM). Reported error with nasal only breathing during normal breathing conditions ranged from 0.0 mmHg at 0 lpm (Oxy2Pro) to 22.1 mmHg at 15 lpm (POM). Higher error was reported across all products during shallow breathing conditions compared to normal breathing conditions. Reported error with oral breathing during shallow breathing conditions ranged from 3.7 mmHg at 0 lpm (Oxy2Pro) to 24.5 mmHg at 15 lpm (Capnovue®). Reported error with nasal breathing during shallow breathing conditions ranged from 3.3 mmHg at 0 lpm to 24.3 mmHg at 15 lpm (OxyPro2). Notably, greater error was reported with PetCO_2_ masks and procedural bite blocks compared to oral-nasal cannula sampling lines.

### Filter performance

**Fig. 5:**
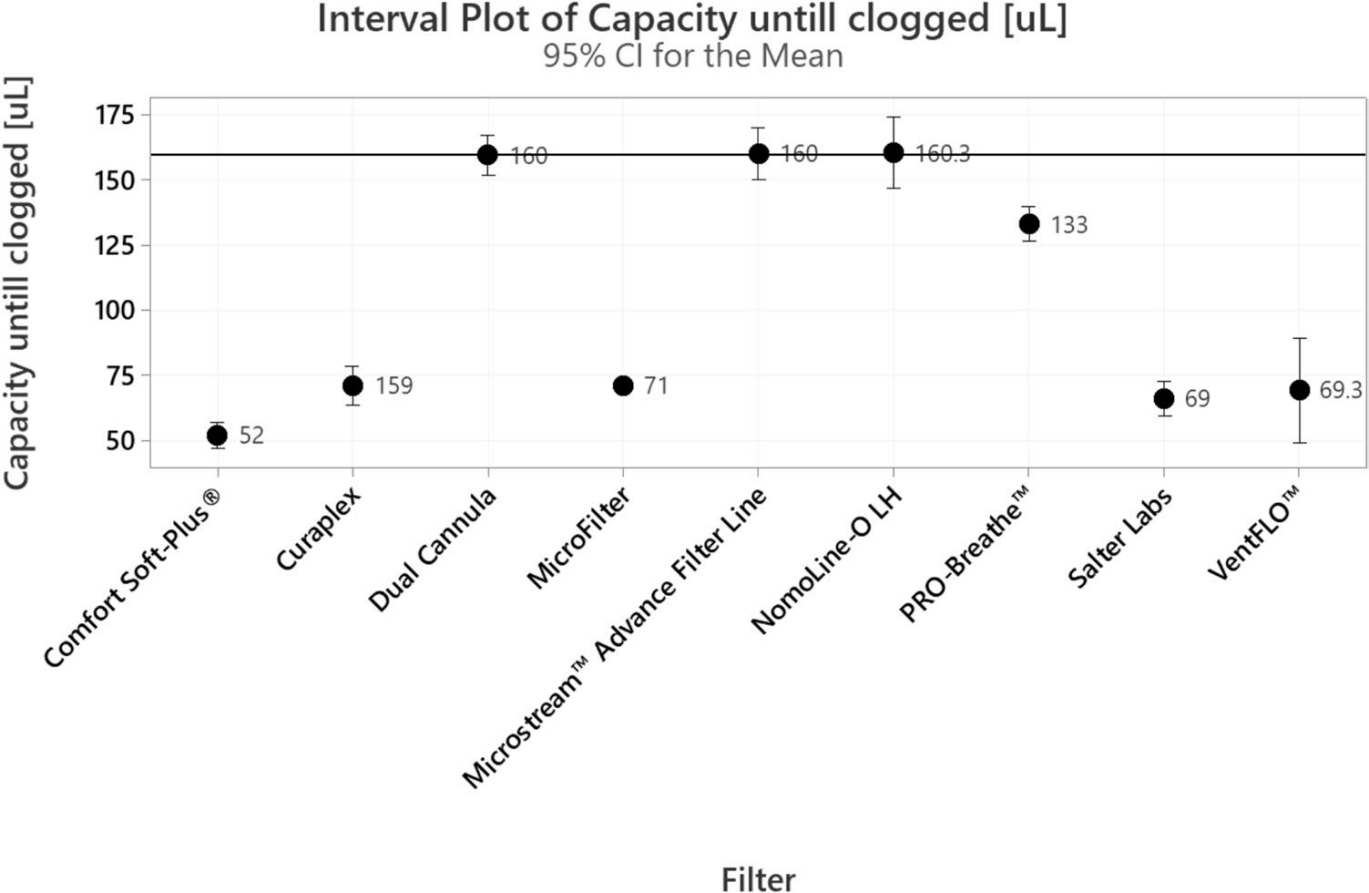
Filter performance, as measured by amount of liquid added to system before clogging of filter was detected, tested up to 160 μL. Flowrate of liquid was set to 1 ml per minute. The black line represented the expected clogging capacity per specifications of the capnography system under study

Figure 5 depicts the filter performance for each of the nine oral-cannula product lines tested. The match-paired (Microstream™ Advance Filter Line) and two cross-paired (Flexicare Dual Cannula, and NomoLine-O LH) oral-nasal cannula filters passed the clog test. The remaining five cross-paired filters appeared clogged prior to the expected threshold of 160 μL, with four of them (MicroFilter, VentFLO™, Salter Labs, Comfort Soft-Plus®) demonstrating a clog capacity of less than half the expected value.

### Mechanical durability

Except for one of the cross-paired oral-nasal cannulas (Salter Labs®), all product lines passed the tensile/pull test remaining intact when pulled with a force of up to 2 kg. During leak testing, one cross-paired oral-nasal cannula product line (PRO-Breathe™) demonstrated leaks suggestive of decreased mechanical integrity, showing improper sealing at both sampling line and O_2_ delivery line.

## Discussion

This study provides evidence of variation in performance of currently available sidestream capnography sampling lines claiming compatibility with Microstream™ enabled capnography monitoring systems. Of the cannulas tested, the match-paired sampling line consistently provided accurate readings, expected filter performance, and mechanical integrity. The cross-paired cannulas were mixed in their performance, suggesting that these cannulas may not provide reliable and/or accurate measurements when used in tandem with a Microstream™ enabled capnography system. Moreover, products that clogged at water volumes lower than 160μL may require more frequent replacement of sampling lines.

This study supports previous reports of increasing error estimates for PetCO_2_ during oral breathing, with increasing supplemental oxygen flow rates, and during conditions of hypoventilation [29, 36, 37]. Notably, error estimates greater than 30 mmHg during oral breathing at an oxygen flow rate of 5 lpm were demonstrated with procedural bite blocks and error estimates greater than 20 mmHg were demonstrated in PetCO_2_ masks during oral breathing when supplemental oxygen flow rates reached 15 lpm. Additionally, this study documented clinically notable PetCO_2_ error estimates (29.1–30.8 mmHg) during conditions of nasal obstruction in split design cannulas. Although higher PetCO_2_ error estimates under conditions of oral breathing, nasal obstruction, administration of supplemental oxygen, and hypoventilation are well documented, accurate capnography monitoring within the limits of clinical acceptability is achievable through careful product selection.

A key clinical value of capnography monitoring lies in early detection of respiratory compromise and prevention of serious respiratory decompensation that may lead to serious adverse events [1, 3, 38]. For a long time, clinical practice has focused on identifying low or high respiratory rates, as these are easily observable signs even without a monitor. However, relying only on respiratory rate means problems are often detected relatively late. Exact values provided by capnography, such as PetCO_2_ levels, allow for much earlier recognition of clinical deterioration before changes in respiratory rate become apparent. This is a key advantage of capnography monitoring beyond simply detecting bradypnea or tachypnea. Patients in post-operative and acute care settings have varying risk profiles for respiratory decompensation. Patients at higher risk for respiratory compromise often present with tachypnea, shallow breathing, and clogged nostrils (e.g., from placement of nasogastric tubes). Therefore, the error rates reported for some product lines during these simulated conditions could have clinical implications. Erroneous readings during episodes of tachypnea and shallow breathing potentially delays the detection of respiratory compromise and may result in serious adverse events.

Though an important factor in this discussion, a cost-analysis was not undertaken, as it was outside the scope of the study. Therefore, information required to evaluate the value per cost for each product line was unable to be computed. Further research in this domain would be fitting to provide more insight. However, recent models suggest that cost savings is more important than actual supply cost in the cost–benefit analysis of capnography [39]. Khanna et al. estimated cost savings for the use of continuous capnography in low, intermittent, and high-risk respiratory depression groups in a cohort of general care patients receiving opioids in a US hospital. Estimates for risk of respiratory compromise, incidence of respiratory compromise, length of stay, capnography supply costs and total hospital admission costs were derived from the PRODIGY Trial [38]. Total hospital costs decreased when continuous capnography monitoring was applied to the high-risk only group, the high and intermediate-risk groups, and all groups combined. The cost savings associated with continuous capnography was significant across three groups: $535,531 with high-risk only patients, $606,463 with high and intermediate risk patients, and $688,221 for all patients. Break-even estimates were computed as the percent reduction in respiratory depression needed to offset the cost of continuous monitoring. The break-even estimates were 1.5% for high-risk patients, 2.5% for high and intermediate-risk patients, and 3.5% for all patients.

Study findings have important implications for clinicians caring for patients at risk for respiratory depression. Clinicians should be aware of the conditions associated with higher errors in PetCO_2_ and be reminded that though capnography serves as a key adjunctive monitoring technology it is not to be used to the exclusion of good physical assessments. Study findings also have implications for supply chain managers and anesthesia providers engaged in capnography product evaluation. Available product lines and manufacturers should not be considered interchangeable. As this study demonstrated, there can be significant differences in performance. Moreover, this study demonstrated the significance of the type and quality of sampling lines in the accuracy of capnography. In this study, all sampling lines were connected to the same capnography monitor. Findings suggest that the substitution of sampling lines from other manufacturers may impact the accuracy and reliability of the capnography system due to longer than expected rise times, reduced filter capacity, and/or reduced structural integrity. As the study endpoints looked only at performance outcomes, no definitive comments can be made indicating specific factors related to the materials or construction of each sampling line that may have contributed to the observed differences in performance.

This study has strengths and limitations. This was a bench study with respiratory rates and volumes simulated by a machine. All conditions were carefully controlled for absolute consistency across product lines. While the use of simulated ventilation conditions enhances the internal validity of the study, it limits external validity. This controlled environment did not capture the nuances present in the complex and dynamic clinical environments in which capnography monitoring is applied. While ventilation was simulated, gas diffusion was not. While different breathing patterns were simulated during testing, some concerning breathing patterns, such as periodic breathing, were not addressed. Moreover, a range of pathologies that potentially affect PetCO_2_ monitoring may not have been reflected in the simulation settings. In contrast to mobile patients, all equipment was stationary. Assessment of errors in PetCO_2_ across available product lines under conditions that more precisely reflect the clinical environment could further support the product evaluation process.

## Conclusion

In conclusion, the results of this bench study suggests that substitution of sampling lines outside the Microstream™ family, may disrupt accuracy of readings when using a Microstream™ enabled capnography monitor.

## Supplementary Information

Below is the link to the electronic supplementary material.

Supplementary file1 (DOCX 1349 kb)

Supplementary file2 (DOCX 13 kb)

## Data Availability

Aggregate data is provided within the manuscript and supplementary information files. Detailed data are available upon reasonable request.

## Abbreviations

IR: Infrared radiation
PetCO_2_: End-tidal carbon dioxide
bpm: Breaths per minute
CO_2_: Carbon dioxide
IFU: Instructions for use
lpm: Liters per minute
ISO: The International Organization for Standardization
