# Supplementary material for: Evaluation of accuracy, filter performance, and durability among capnography sampling lines: a bench study
Source: J Clin Monit Comput. 2025 Aug 28;40(1):81–92. doi: 10.1007/s10877-025-01346-3 (PMC12963109; doi:10.1007/s10877-025-01346-3)
Supplement: Supplementary file 1 — Supplementary file1 (DOCX 1349 kb) [file 10877_2025_1346_MOESM1_ESM.docx]

**Supplementary Figure 1.** Sample waveforms of two oral-nasal cannulas at breathing rates between 10 and 80 bpm. The Microstream™ Advance Filter Line (rise time = 157.3 milliseconds) is depicted in the dark blue tracings, the PRO-Breathe™ (rise time = 273 milliseconds) is depicted in light blue, and the Flexicare Dual Cannula (rise time = 689.7 milliseconds) is depicted in the brown tracings. For this study set-up, an expected single breath tracing would read 36 mmHg at the peak of the breath and 0 mmHg at the end of the breath, regardless of breathing rate, as is seen in the Microstream Advance Filter Line. As breathing rate increases, the tracing of the PRO-Breathe and Flexicare Dual Cannula begin to read below the expected 36 mmHg and the end of the breath does not reach the expected 0 mmHg. This illustrates how rise times outside the specifications of the capnography system can lead to inaccurate PetCO_2_ measures.
