# Supplementary material for: Evaluation of accuracy, filter performance, and durability among capnography sampling lines: a bench study
Source: J Clin Monit Comput. 2025 Aug 28;40(1):81–92. doi: 10.1007/s10877-025-01346-3 (PMC12963109; doi:10.1007/s10877-025-01346-3)
Supplement: Supplementary file 2 — Supplementary file2 (DOCX 13 kb) [file 10877_2025_1346_MOESM2_ESM.docx]

Supplementary Table 1. Overview of testing results for oral-nasal cannulas

| **Sampling Line**  **Manufacturer** | **Rise Time** | **EtCO_2_ Accuracy**  **10 – 80 bpm** | **EtCO_2_ Accuracy**  **Nasal** | **EtCO_2_ Accuracy**  **Nasal Obstructed** | **EtCO_2_ Accuracy**  **Nasal with O_2_** | **EtCO_2_ Accuracy**  **Oral Breathing** | **Filter Performance** | **Pull Test** | **Leak Test** |
| --- | --- | --- | --- | --- | --- | --- | --- | --- | --- |
| *Microstream™ Advance Filter Line*  *Medtronic* | Within Specifications | Within Specifications | Within Specifications | Within Specifications | Within Specifications | Within Specifications | Within Specifications | Within Specifications | Within Specifications |
| *Salter Labs* | **Outside Specifications** | **Outside Specifications** | Within Specifications | **Outside Specifications** | Within Specifications | Within Specifications | **Outside Specifications** | **Outside Specifications** | Within Specifications |
| *Comfort Soft-Plus®*  *WestMed* | **Outside Specifications** | **Outside Specifications** | Within Specifications | **Outside Specifications** | Within Specifications | **Outside Specifications** | **Outside Specifications** | Within Specifications | Within Specifications |
| *NomoLine-O LH*  *Masimo* | **Outside Specifications** | Within Specifications | Within Specifications | **Outside Specifications** | Within Specifications | **Outside Specifications** | Within Specifications | Within Specifications | Within Specifications |
| *PRO-Breathe™*  *PROACT* | **Outside Specifications** | **Outside Specifications** | Within Specifications | **Outside Specifications** | Within Specifications | Within Specifications | Within Specifications | Within Specifications | **Outside Specifications** |
| *VentFLO™*  *SunMed®* | **Outside Specifications** | **Outside Specifications** | Within Specifications | Within Specifications | Within Specifications | Within Specifications | **Outside Specifications** | Within Specifications | Within Specifications |
| *Curaplex^1^* | **Outside Specifications** | **Outside Specifications** | Within Specifications | Within Specifications | Within Specifications | Within Specifications | **Outside Specifications** | Within Specifications | Within Specifications |
| *MicroFilter*  *MedLine* | **Outside Specifications** | **Outside Specifications** | Within Specifications | Within Specifications | Within Specifications | Within Specifications | **Outside Specifications** | Within Specifications | Within Specifications |
| *Dual Cannula*  *Flexicare* | **Outside Specifications** | **Outside Specifications** | Within Specifications | Within Specifications | **Outside Specification**s | Within Specifications | Within Specifications | Within Specifications | Within Specifications |

“Outside specifications” was defined as measurements that fell outside the specifications defined in the instructions for use for the capnograph system used for testing. If multiple conditions were tested, falling outside specifications for any condition was marked as “outside specifications”.
